# Estimating changes in life expectancy in Hong Kong during the COVID-19 pandemic: a longitudinal ecological study

**DOI:** 10.1016/j.lanwpc.2025.101571

**Published:** 2025-05-08

**Authors:** Alexandra H.T. Law, Anne M. Presanis, Justin K. Cheung, Peng Wu, C. Mary Schooling, Benjamin J. Cowling, Jessica Y. Wong

**Affiliations:** a World Health Organization Collaborating Centre for Infectious Disease Epidemiology and Control, School of Public Health, The University of Hong Kong, Pokfulam, Hong Kong Special Administrative Region of China; b MRC Biostatistics Unit, University of Cambridge, Cambridge, United Kingdom; c School of Public Health, The University of Hong Kong, Pokfulam, Hong Kong Special Administrative Region of China; d Laboratory of Data Discovery for Health Limited, Hong Kong Science and Technology Park, New Territories, Hong Kong Special Administrative Region of China

**Keywords:** COVID-19, Life expectancy, Life table, Cause of death, Uncertainty, Parametric bootstrap

## Abstract

**Background:**

Hong Kong has one of the longest life expectancies in the world but was heavily impacted by COVID-19 in 2022. We aimed to estimate patterns in mortality rates and changes in life expectancy in Hong Kong during the COVID-19 pandemic.

**Methods:**

We constructed sex-specific life tables from 1998 to 2023 using parametric bootstrapping to account for statistical uncertainty in mortality rates. We used Arriaga’s decomposition method to estimate age- and cause-specific contributions to overall changes in life expectancy for 2020–2023, with 2019 as the reference year. We also estimated cause-specific mortality rates.

**Findings:**

Hong Kong reported 50,666 deaths in 2020, 51,354 in 2021, 63,692 in 2022, and 54,731 in 2023. Estimates of life expectancy in males and females in 2020 and 2021 were similar to the pre-pandemic trend from 1998 to 2019 but declined significantly in 2022. Compared to the pre-pandemic trend, the 2022 values of 80.4 years for males and 86.4 years for females corresponded to reductions by 2.22 (95% CI: 2.08, 2.36) years in males and 2.30 (95% CI: 2.17, 2.43) years in females. The loss in life expectancy in 2022 was mainly attributed to increased respiratory mortality rates, with a negative contribution to life expectancy of 1.47 and 1.26 years for males and females respectively. In 2023 life expectancy increased by 0.60 (95% CI: 0.46, 0.75) years in males and by 1.10 (95% CI: 0.95, 1.26) years in females.

**Interpretation:**

In 2022 a very high respiratory mortality rate in older adults in Hong Kong during the COVID-19 pandemic was associated with a reduction in life expectancy by more than 2 years. In 2023 life expectancy increased towards the pre-pandemic trend.

**Funding:**

10.13039/501100005847Health and Medical Research Fund, Hong Kong.

**Research in context**

**Evidence before this study**

The Coronavirus disease (COVID-19) pandemic has resulted in more than 7 million deaths globally since 2020, with an estimated decrease in global life expectancy of 1.6 years from 2019 to 2021. A drop in life expectancy has been reported in many places during the pandemic, including locations in Asia such as Japan and South Korea. The Hong Kong Special Administrative Region of the People’s Republic of China had one of the highest life expectancies in men and women before the pandemic, but official statistics indicated a substantial drop in life expectancy in 2022. To understand the change in life expectancy associated with COVID-19 different populations in Asia, we searched PubMed to 24 July 2024 using the following search terms: “(“SARS-CoV-2” OR “COVID-19”) AND (“life expectancy”)”. We identified one study investigating the impacts on life expectancy in Asia during 2019–2021 which suggested increased mortality in older adults aged 60 years or above contributed most to the reduction of life expectancy. Another research studying the rural-urban gap in life expectancy in China in 2020–2021 suggested a wider gap in life expectancy during the pandemic with a shifting trend towards older ages and the population having circulatory diseases. A study in South Korea found that life expectancy was reduced in 2020 and 2021 with increased mortality rates in older adults. Another study reported that life expectancy in Japan was slightly shortened by 0.15 years in 2020–2021, and females (0.15 years) experienced slightly larger losses than males (0.12 years). The losses in life expectancy were mainly attributable to increased mortality in older adults aged 70 years or above. However, mortality associated with neoplastic tumors and cardiovascular diseases contributed to life expectancy loss, whereas respiratory diseases did not appear to contribute to the change in Japan, in 2020–2021.

**Added value of this study**

This is the first study to analyze the change in life expectancy at different stages of the pandemic compared to the pre-pandemic trend and to discuss the contributors to this change in Hong Kong. We estimated that life expectancy for males was 82.3 years in 2021 and 80.4 years in 2022, and for females, it was 87.9 in 2021 and 86.4 years in 2022. Reductions of 2.22 (95% CI: 2.08, 2.36) years in males and 2.30 (95% CI: 2.17, 2.43) years in females were observed compared to the pre-pandemic trend. The drop in life expectancy in 2022 was mainly associated with increased respiratory mortality in the population aged 65 or above. There were increases in life expectancy of 0.60 (95% CI: 0.46, 0.75) years in males and of 1.10 (95% CI: 0.95, 1.26) years in females, in 2023, which were still below the extrapolated pre-pandemic trend due to the increased mortality rates from respiratory and cardiovascular diseases. There was no evidence of substantial mortality displacement from other causes.

**Implications of all the available evidence**

The substantial decrease in life expectancy in Hong Kong in 2022 was associated with increased respiratory mortality among older adults during a large epidemic of SARS-CoV-2 Omicron BA.2, emphasizing the importance of achieving a high vaccination rate and implementation of protective measures targeting vulnerable older adults. The significant impact of COVID-19 on population health, as indicated by life expectancy, underscored the significance of resource allocation in policymaking.

## Introduction

Life expectancy from birth is a common measure used for international comparisons of population health.^1^ The infant mortality rate, prevalence of chronic diseases, and economic growth, have a significant impact on life expectancy. In Hong Kong, advances in healthcare, driven by successful economic development, have resulted in a remarkable increase in life expectancy in recent decades, making it one of the longest globally.^2^ This achievement is attributed to a low infant mortality rate of 1.7 per 1000 live births in 2021,^3^ potentially healthy migrant effects,^4^ low smoking rates particularly among women,^2^ few deaths from transport accidents,^2^ significant investments in public healthcare,^2^ and possibly overstatement of age in older people due to use of Chinese age (one year old at birth and two years old at first Chinese New Year) coupled with lack of birth certificates. However, the rising prevalence of chronic diseases and multimorbidity among older adults complicates health outcomes and life expectancy trends. A decrease in life expectancy was observed in several Asian countries with high life expectancies in 2022 after the spread of Omicron, including China,^5^ Japan,^6^, ^7^, ^8^ Singapore,^9^ and South Korea.^10^^,^^11^

Hong Kong used public health and social measures to minimize COVID-19 transmission for the first two years of the pandemic,^12^ but experienced a major epidemic of Omicron BA.2 in early 2022 resulting in around half of the population being infected and more than 10,000 confirmed COVID-19 deaths among Hong Kong’s 7.3 million population.^13^^,^^14^ The majority of the deaths were observed in older adults ≥65 years of age.^15^ The reported number of COVID-19 deaths might not reveal the full picture of the impact of the pandemic because some confirmed deaths might have occurred with COVID-19 rather than because of COVID-19, while the control measures used to reduce transmission also had a substantial indirect impact which may have affected mortality.^16^ Distinguishing whether adverse health impacts stem from the pandemic itself or the control measures is important for understanding the sources of mortality impacts and analyzing the relationship between public health policies and changes in life expectancy. The World Health Organization estimated that 14.83 million excess deaths occurred in the first two years of the pandemic.^17^ The Institute for Health Metrics and Evaluation estimated that global life expectancy decreased by 1.6 years from 2019 to 2021,^18^ which was a dramatic reversal from the slowly increasing trend before 2019.

To explore how the COVID-19 pandemic affected mortality in Hong Kong, a city with a high proportion of older adults, in this study we estimated patterns in mortality rates and the consequent life expectancy changes in Hong Kong during the COVID-19 pandemic.

## Methods

### Sources of data

Age-specific weekly deaths from 1998 to 2023 were obtained from the Census and Statistics Department of the Government of the Hong Kong Special Administrative Region. For analysis of cause-specific death rates, we selected 9 major primary causes of death for both sexes in Hong Kong since 2001, including malignant neoplasms, cardiovascular, respiratory, kidney diseases, diabetes mellitus, chronic liver disease, dementia, septicemia, and external causes, which has observed a higher extent of increase among other leading causes in 2022. A final category, “other causes”, included all other deaths. These ten cause of death groupings were coded according to the International Classification of Diseases, Ninth Revision (ICD-9) from 1998 to 2000 and Tenth Revision (ICD-10) from 2001 onwards (Appendix). Almost all deaths in Hong Kong occur in hospital, which facilitates accurate coding. The age-specific mid-year population sizes from 1998 to 2023 were obtained from the Census and Statistics Department and were used as the denominators for the estimation of mortality rates.^19^ The population sizes for ages 85 to ≥100 years in 1996, 2001, 2006, 2011, 2016, and 2021 were obtained from the censuses and by-censuses conducted by the Census and Statistics Department.^20^ These data were used to estimate the population size from age 85 to ≥100 years from 1998 to 2023 (Appendix). Our study received ethical approval from the Institutional Review Board of the University of Hong Kong (UW 21–393). Participant consent was not required for this longitudinal ecological study which used aggregated and deidentified mortality data and census data on population sizes.

### Statistical analysis

We constructed life tables for each year from 1998 to 2023 based on local mortality rates by age and sex (Appendix). We used a parametric bootstrap approach to estimate uncertainty in the mortality rates by repeatedly sampling from fitted negative binomial distributions for observed death counts. We estimated the value of the overdispersion parameter alpha by fitting the negative binomial regression model to the mortality data. We estimated standardized mortality rates for males and females from 1998 to 2023 using the respective population structures in 2019 as the reference populations. We identified the best-fitting and most parsimonious regression models for the change in life expectancy between 1998 and 2019 and extrapolated this linear fit to the pandemic years.

Arriaga’s decomposition method was used to investigate the contribution of different age groups and causes of death to changes in life expectancy in 2020, 2021, 2022, and 2023, compared to the reference year of 2019.^21^ The age-specific contribution consists of both direct and indirect effects. However, the last age group only gives a direct contribution, as there is no older age constituting an indirect effect. The sum of effects from all ages and the total change in life expectancy in years should be equal. The change in life expectancy by age was estimated for six age groups: 0–4, 5–14, 15–44, 45–64, 65–79, and ≥80 years. We used 2019 as the reference year for this decomposition analysis rather than from one year to the next during the COVID-19 pandemic because the increased mortality rates in the pandemic would affect the comparisons in later years. Using 2019 as a baseline to compare this with COVID-19 years is intended to reflect the changes in mortality patterns in the pandemic versus in the pre-pandemic periods. More computational details of Arriaga’s decomposition are provided in the Appendix. All statistical analyses were conducted in R version 4.3.3 (R Foundation for Statistical Computing, Vienna, Austria).

### Role of the funding source

The funding bodies had no role in the design of the study, the collection, analysis, and interpretation of data, or writing of the manuscript.

## Results

**Fig. 1:**
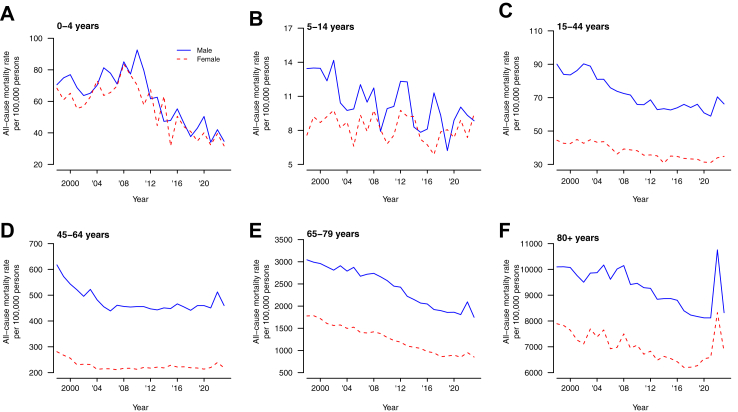
All-cause mortality rates in Hong Kong by age and sex, from 1998 to 2023. Panel A: age 0–4 years. Panel B: age 5–14 years. Panel C: age 15–44 years. Panel D: 45–64 years. Panel E: 65–79 years. Panel F: 80+ years.

**Fig. 2:**
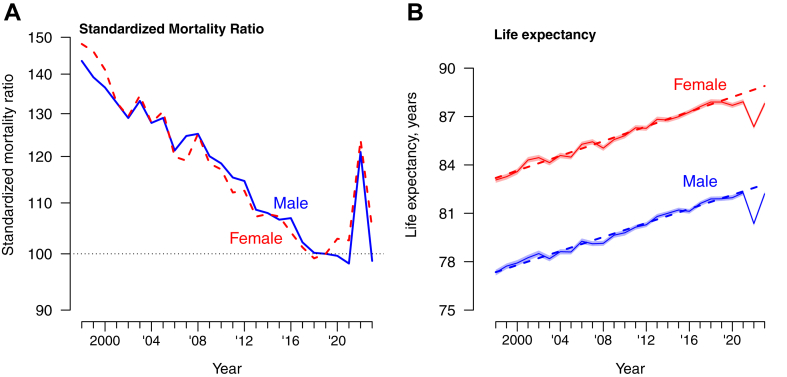
Panel A: Standardized mortality ratios for males and females in Hong Kong compared to the reference year of 2019. Panel B: Life Expectancy for males and females in Hong Kong from 1998 to 2023 with 95% confidence intervals. The dashed lines show the fitted linear trends from 1998 to 2019 extrapolated to 2020–2023.

Fig. 1 shows the all-cause mortality rates from 1998 to 2023 for males and females in six age groups. From 1998 through to 2021 the age-specific mortality rates generally declined over time for both males and females. The mortality rates increased substantially in 2022 in all age groups except in 5–14 years. In particular, the estimated mortality rates among males and females ≥80 years increased dramatically from 2021 to 2022. For males, mortality rates increased from 8120 per 100,000 in 2021–10,800 per 100,000 in 2022, and for females it increased from 6590 per 100,000 in 2021–8330 per 100,000 in 2022. The standard mortality rates for males and females gradually declined over time but increased drastically in 2022, from 100 (reference) in 2019, to 121 and 124 for males and females respectively (Fig. 2A).

The long-term decreases in mortality rates over the study period resulted in a steady increase in life expectancy for both sexes, from 77.3 in 1998 to 82.3 in 2021 for males, and from 83.1 in 1998 to 87.9 in 2021 for females (Fig. 2B). In 2022, life expectancy declined to 80.4 years in males and 86.4 years in females. Using linear regression models, we projected the trend in life expectancy from 1998 through 2019 into the three pandemic years, shown as dotted lines in Fig. 2B. The autocorrelation with the Durbin–Watson Test for the male and female models was 1.60 and 2.04 respectively, indicating that there is little to no autocorrelation in the residuals of both models, and more complex models did not provide improved fit for the trends in the data from 1998 to 2019. Compared to the pre-pandemic secular trends in life expectancy for males and females, life expectancy decreased slightly in 2020 and 2021 in females while it remained similar to the projected trend in males in those two years. Life expectancy then declined very substantially in 2022 by 2.22 (95% confidence interval, CI: 2.08, 2.36) years in males and by 2.30 (95% CI: 2.17, 2.43) years in females, compared to the extrapolated value from the slowly increasing trend from 1998 to 2019.

**Fig. 3:**
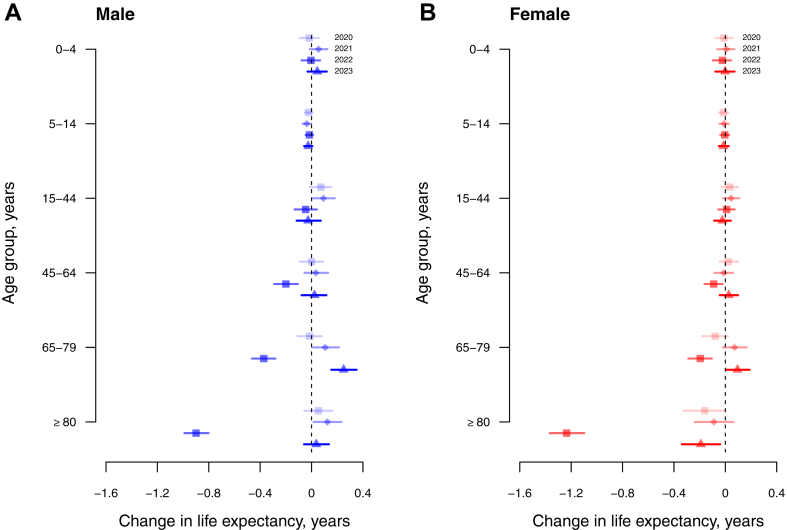
Arriaga decomposition of age-specific contributions (with 95% confidence intervals) to changes in life expectancy in 2020 (light-shaded squares), 2021 (diamonds), 2022 (dark-shaded squares), and 2023 (triangle) for males (Panel A) and females (Panel B), compared to the reference year 2019. Each estimate shows the positive or negative change in life expectancy in the corresponding age group versus the pattern observed in 2019. Values of zero would correspond to no net change in life expectancy attributable to that age group during the specified period compared to the reference year of 2019.

In 2023, life expectancy rose in males and females back towards the pre-pandemic trend, increasing by 0.60 (95% CI: 0.46, 0.75) years in males and by 1.10 (95% CI: 0.95, 1.26) years in females. The life expectancy estimates of 82.2 (95% CI: 82.1, 82.3 years in males and 87.8 (95% CI: 87.6, 87.9) years in females were still lower than the pre-pandemic trend which under our simple linear extrapolation would have corresponded to life expectancies of 82.8 years in males and 88.9 years in females in 2023 (Fig. 2B). In our decomposition analysis by age group, the statistically significant reduction in female life expectancy from 2019 to 2022 was largely attributed to the increase in mortality rate among females aged 80 years or above, followed by those aged 65–79 years (Fig. 3). Similarly, the decrease in male life expectancy from 2019 to 2022 was also attributed to increased mortality rates in males aged ≥80 years, followed by those 65–79 years.

**Fig. 4:**
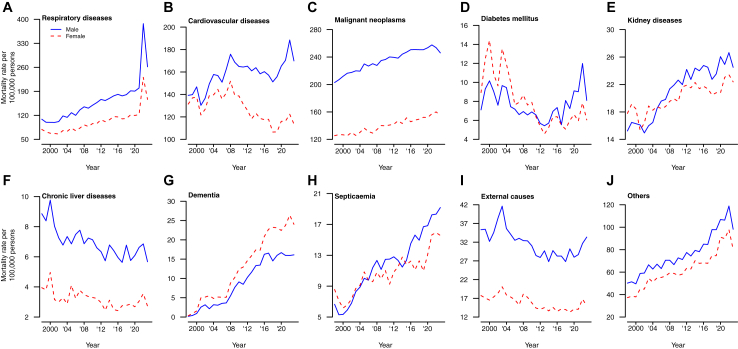
Mortality rates in Hong Kong by cause and by sex from 1998 to 2023. Panel A: Respiratory diseases. Panel B: Cardiovascular diseases. Panel C: Malignant neoplasms. Panel D: Diabetes mellitus. Panel E: Kidney diseases. Panel F: Chronic liver diseases. Panel G: Dementia. Panel H: Septicaemia. Panel I: External causes. Panel J: Others.

Fig. 4 shows the mortality rates by cause and by sex from 1998 to 2023. The largest increase in 2022 was observed in mortality from respiratory causes, and smaller increases in mortality rates were observed in 2022 in several other cause of death categories, except cancer for males, in 2022. From 1998 to 2023, most of the cause-specific mortality rate of males were higher than that of females, apart from diabetes, kidney diseases, and septicemia. In 2023, cause-specific mortality rates returned to levels that were comparable to those prior to the COVID-19 pandemic for some causes although respiratory and cardiovascular mortality remained elevated.

**Fig. 5:**
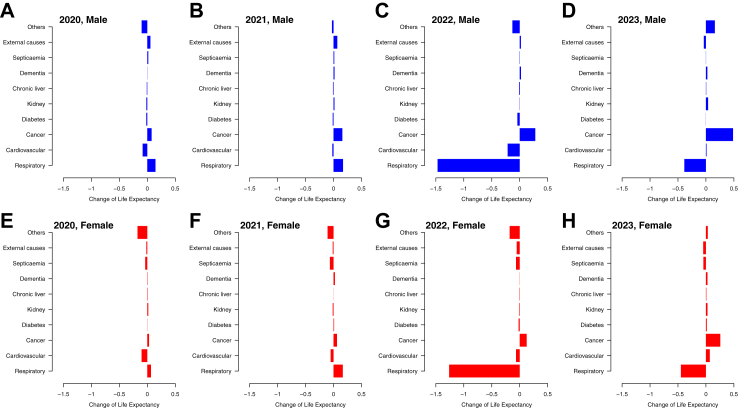
Arriaga decomposition of cause-specific contributions to changes in life expectancy in 2020, 2021, 2022 and 2023 for males and females, compared to the reference year 2019. Each estimate shows the positive or negative change in life expectancy in the corresponding causes of death versus the pattern observed in 2019. Panel A: males, 2020. Panel B: males, 2021. Panel C: males, 2022. Panel D: males, 2023. Panel E: females, 2020. Panel F: females, 2021. Panel G: females, 2022. Panel H: females, 2023.

Substantial increases in mortality rates from respiratory causes was the main driver of the decrease in life expectancy in 2022 for both sexes in 2022, contributing to reductions of −1.47 years for males and −1.26 years for females (Fig. 5). Cancer was the only cause of death in which changes were associated with an increase in life expectancy in 2022 rather than a decrease. Increased mortality from respiratory causes was still a major contributor to reduced life expectancy in 2023 compared to 2019, but with smaller extent compared to the preceding year (Fig. 5).

## Discussion

COVID-19 was successfully contained in Hong Kong in 2020 and 2021, with fewer than 1% of the population infected,^22^ but the uncontrolled spread of Omicron BA.2 in early 2022 had a very substantial impact.^13^^,^^23^ The loss in life expectancy in 2022 compared to an extrapolated prediction based on the pre-pandemic trend, by 2.22 years in males and by 2.30 years in females, was mainly attributed to a large increase in mortality rates in persons aged ≥65 years (Fig. 3). In cause-specific analysis we identified increases in mortality from respiratory causes and cardiovascular causes (Fig. 4). There was no evidence of substantial displacement of mortality from other causes. In 2023, life expectancy in Hong Kong increased towards the pre-pandemic trend (Fig. 2), but mortality rates remained elevated for respiratory diseases and cardiovascular diseases (Figs. 4 and 5) and male and female life expectancy remained somewhat below the extrapolated pre-pandemic trend.

The high mortality impact of SARS-CoV-2 Omicron BA.2 in Hong Kong in early 2022 has been discussed at length elsewhere, and can be attributed to lower COVID-19 vaccine coverage in older adults and severe strain on healthcare resources in March and April 2022.^13^^,^^24^^,^^25^ By early 2022 the SARS-CoV-2 vaccination rate among individuals aged 80 years or above was the lowest among all adult age groups in Hong Kong, with 20.5% of males and 17.1% of females in this age group receiving at least two doses of SARS-CoV-2 vaccination at the beginning of 2022, compared to over 80% for adults ≥18 years overall.^12^^,^^26^^,^^27^ The SARS-CoV-2 mortality rate was particularly high among older adults in residential care. The explosive spread of infections in early 2022 caused severe strain on healthcare resources leading to a three-fold increase in case fatality rates at the peak of the epidemic in March compared to earlier or later in that epidemic wave.^24^ In addition, the strain on healthcare resources resulting from the large number of SARS-CoV-2 cases and respiratory hospitalizations likely had negative consequences for cardiovascular services and disrupted referrals, diagnoses, and timely interventions.^25^ Banerjee at el. Reported that substantial excess mortality burden in cardiovascular causes was discovered in China, Italy, and the United Kingdom during the COVID-19 pandemic due to disruptions in medical services for patients with cardiovascular diseases.^28^ The Hong Kong government implemented stringent containment policies to slow down the spread of the Omicron virus in the community in January–April 2022, but a large peak of infections and deaths was still recorded in March 2022, highlighting the challenges of controlling this highly transmissible variant.^12^ Daily COVID-19 case numbers dropped temporarily in late-May 2022, but resurged again in June with a wave of BA.4/5 infections.

The mortality rate for diabetes mellitus increased significantly in 2022 and dropped back to the baseline level in 2023. Wan et al. reported that COVID-19 has been associated with an increase in deaths related to diabetes complications, possibly because of delays or disruptions in routine care.^29^ In addition, the gap between the sexes in cardiovascular deaths was more pronounced in 2022 with more cardiovascular deaths in males. This difference may be attributed to a higher prevalence of cardiovascular diseases in males.^30^

In contrast to previous findings on excess mortality which reflect immediate burden of disease,^23^^,^^25^ our analysis of life expectancy captures the broader shifts in mortality in a population in a similar way to a “years of life lost” assessment in a cohort. Life expectancy is calculated from a life table where the observed age-specific mortality rates are applied to a standard population of one million births, as if the age-specific mortality rates would apply to that cohort as they progress through life. The construction of the life table enables the modeling of mortality rates as if they apply uniformly to all populations in that cohort as they age, regardless of the actual age distribution of the population being studied. We also estimated the standardized mortality ratio to compare the mortality rate of a year-specific population to a standard population. This allows a standardized comparison of age-specific mortality rates among populations with different age structures. In Hong Kong’s ageing population the COVID-19 pandemic caused a very substantial number of deaths in older adults, and the COVID-19 mortality rate in Hong Kong in 2022 was one of the highest per capita rates in the world. The loss of slightly more than two years of life expectancy in both males and females during the COVID-19 pandemic in Hong Kong implies that there were a substantial number of years of life lost. Notably, higher COVID-19 mortality rates in the United Kingdom in 2020 and 2021 (than in Hong Kong in 2022) did not translate to such substantial changes in life expectancy.^31^

Prior to the COVID-19 pandemic, global life expectancy increased from 62 years in 1980 to 72 years in 2015.^18^ Hong Kong has also experienced steady increases in life expectancy over the last 25 years, but the COVID-19 pandemic had a drastic impact on life expectancy in 2022 (Fig. 2). COVID-19 likely impacted life expectancy in almost every country, for example the life expectancy of males (females) in the United States decreased significantly by 2.1 (1.5) years in 2020, by a further 0.7 (0.6) years in 2021, and then increased by 1.3 (0.9) years in 2022 to a level still 1.5 (1.2) years below that in 2019.^32^ The United Kingdom reported reductions of life expectancy by 0.1–0.3 years each consecutive year in 2020, 2021 and 2022.^31^ Locations with high COVID-19 mortality rates in 2020 and 2021 would tend to have reductions in estimated life expectancy in those years. In contrast, Hong Kong along with several other locations in the Asia–Pacific region was able to minimize COVID-19 mortality until 2022,^12^ and did not have substantial changes in life expectancy until 2022. Similar to Hong Kong, South Korea had slight increases in life expectancy in 2020 and 2021 but then had a reduction in life expectancy by 0.7 years in males and 1.0 years in females in 2022.^33^ Singapore reported almost no change in life expectancy in 2020, reductions by 0.4 and 0.5 years in males and females in 2021, and by 0.3 and 0.1 years in 2022 respectively.^9^ Australia and New Zealand reported almost no change in life expectancy over the period 2020–2022.^34^^,^^35^ Therefore, Hong Kong appears to have recorded a particularly substantial change in life expectancy compared to other locations.

The substantial reduction in life expectancy in Hong Kong in 2022 could be explained by several factors. Hong Kong’s ageing population means it is more vulnerable to the impact of a respiratory virus pandemic in which severity drastically increases with age. In Hong Kong, the proportion of older adults aged 65 year or above increased from 10.6% in 1998 to 21.8% in 2023.^30^ During the surge in Omicron infections in early 2022, the healthcare system faced unprecedented pressure, which may have affected the provision of care for both COVID-19 and non-COVID-19 patients.^24^ Furthermore, public health policies may have inadvertently resulted in social isolation and reduced access to medical care, impacting health outcomes more broadly in the community.

There are several limitations to discuss. First, we were not able to obtain mid-year population size for ages 85 to ≥100 years, and we made reasonable assumptions about the distribution of population in this range (Appendix). Second, Arriaga’s decomposition method is a common approach to assess changes in life expectancy but it is limited to decomposing the discrete changes such as changes from one year to the next. In Arriaga’s decomposition method, the timing of changes within the time intervals is not captured, and the effects are averaged over the discrete periods. Third, we were not able to separate the direct impact, referring to deaths from COVID-19, from the indirect impact, referring to the consequences of intervention policies implemented during the pandemic for example delays in ambulances responding to heart attacks, or increases in underlying medical conditions due to social isolation, change of lifestyles and delay of healthcare seeking behaviors, etc. We previously reported an increase in cardiovascular mortality in 2020 likely attributable to changes in healthcare seeking behaviors.^16^ In a future study it would be of interest to separate the direct and indirect effects of the pandemic by including more covariates, including the effectiveness of the intervention policies, the long-term health impacts resulting from SARS-CoV-2 infections, and changes in access to and utilization of medical services.

In conclusion, we estimated a substantial decrease in life expectancy in males and females in 2022, associated with substantial increases in all-cause mortality rates particularly in males and females aged 80 years or above, followed by those aged 65–79 years, when a substantial drop in life expectancy occurred compared to the gradually increasing trend in the pre-pandemic years. Life expectancy increased somewhat in 2023 although it did not return to the pre-pandemic trend.

## Contributors

All authors meet the ICMJE criteria for authorship. Conceived and designed the analysis: BJC and JYW; Collated and curated the data: AHTL; Performed the statistical analysis: AHTL; Supervised the analysis: BJC and JYW. Wrote the first draft of the manuscript: AHTL. All authors provided critical review and revision of the text and approved the final version. Jessica Y. Wong verified the data. Alexandra H. T. Law, Justin K. Cheung, Peng Wu, Benjamin J. Cowling and Jessica Y. Wong had access to raw data. Jessica Y. Wong had final responsibility for the decision to submit for publication.

## Data sharing statement

Restrictions apply to the availability of these data. The mortality data and population data are available from the Census and Statistics Department of the Government of the Hong Kong Special Administrative Region (email: gen-enquiry@censtatd.gov.hk).

## Editor note

The Lancet Group takes a neutral position with respect to territorial claims in published maps and institutional affiliations.

## Declaration of interests

B.J.C. has consulted for AstraZeneca, Fosun Pharma, GlaxoSmithKline, Haleon, Moderna, Novavax, Pfizer, Roche, and Sanofi Pasteur. All other authors report no potential conflicts of interest.
